# From vision to vital signs: uncovering severe Anemia through retinal clues!

**DOI:** 10.1093/omcr/omaf305

**Published:** 2026-02-18

**Authors:** Aswini Devi S B, Nishant Yadav, Shaifali Khandpur, Shilpa Gupta

**Affiliations:** Department of Ophthalmology, Deen Dayal Upadhyay Hospital, Government of NCT of Delhi, Hari Nagar, West Delhi, New Delhi 110064, India; Department of Ophthalmology, Deen Dayal Upadhyay Hospital, Government of NCT of Delhi, Hari Nagar, West Delhi, New Delhi 110064, India; Department of Ophthalmology, Deen Dayal Upadhyay Hospital, Government of NCT of Delhi, Hari Nagar, West Delhi, New Delhi 110064, India; Department of Ophthalmology, Deen Dayal Upadhyay Hospital, Government of NCT of Delhi, Hari Nagar, West Delhi, New Delhi 110064, India

**Keywords:** Anemic retinopathy, megaloblastic anemia, vitamin B 12 deficiency, Roth spots, retinal hemorrhages, pancytopenia

## Abstract

A 28-year-old woman presented in the emergency with sudden, painless bilateral loss of vision over one week. Fundus examination revealed multiple white-centered retinal hemorrhages (Roth spots), cotton wool spots, and multiple retinal hemorrhages in both eyes, indicative of anemic retinopathy. Laboratory evaluation showed profound pancytopenia: hemoglobin 2.3 g/dl, leukocyte count 3.0 × 10^9/l, platelet count 92 × 10^9/l, with elevated mean corpuscular volume (107 fl). Serum cobalamin (vitamin B12) was 57 pg/ml and folate 3.5 ng/ml, consistent with megaloblastic anemia due to vitamin B12 deficiency. Peripheral blood smear confirmed macro-ovalocytes and hypersegmented neutrophils. The patient was managed emergently with packed red blood cell transfusions and parenteral vitamin B 12 and folate supplementation. Two weeks later, her hemoglobin had improved to 10.1 g/dl, and follow-up fundus examination showed marked resolution of retinal hemorrhages and Roth spots with partial visual recovery. This case highlights that severe megaloblastic anemia can present as an acute ophthalmic emergency.

## Introduction

Anemia is a common hematologic disorder that can manifest with retinal signs, especially in severe cases [1]. Anemia-associated retinopathy is reported in up to 20%–28% of patients with severe anemia, particularly when hemoglobin levels fall below 8 g/dl. The typical funduscopic findings include intraretinal hemorrhages across all retinal layers, ‘white-centered’ hemorrhages known as Roth spots, soft exudates (cotton wool spots), and dilated, tortuous retinal veins [2].

Megaloblastic anemia due to vitamin B12 (cobalamin) deficiency is an uncommon cause of severe anemia-associated retinopathy. Vitamin B12 deficiency classically presents with hematologic abnormalities (macrocytic anemia, often with pancytopenia) and neurologic features, and occasionally optic neuropathy, but retinal hemorrhages are rarely reported [3]. We present a case of a young woman in whom sudden bilateral vision loss led to the diagnosis of profound megaloblastic anemia due to vitamin B12 deficiency. This case underlines the importance of ophthalmic examination in revealing systemic hematological disease, and the reversibility of ocular findings with timely treatment.

## Case report

A 28-year-old female student, with no prior medical history, presented to the emergency department with diminution of vision in both eyes for 5–6 days. The vision loss was acute in onset, painless, and non-progressive after its initial deterioration. The patient also reported extreme fatigue, generalized weakness, over the past week along with paraesthesia over extremities. There was a history of menorrhagia with menstrual periods of 15 days with a brief inter-menstrual gap of 20 days. She adhered to a strict vegetarian diet and there was no history of diabetes, hypertension, bleeding disorders, or alcohol use.

**Figure 1:**
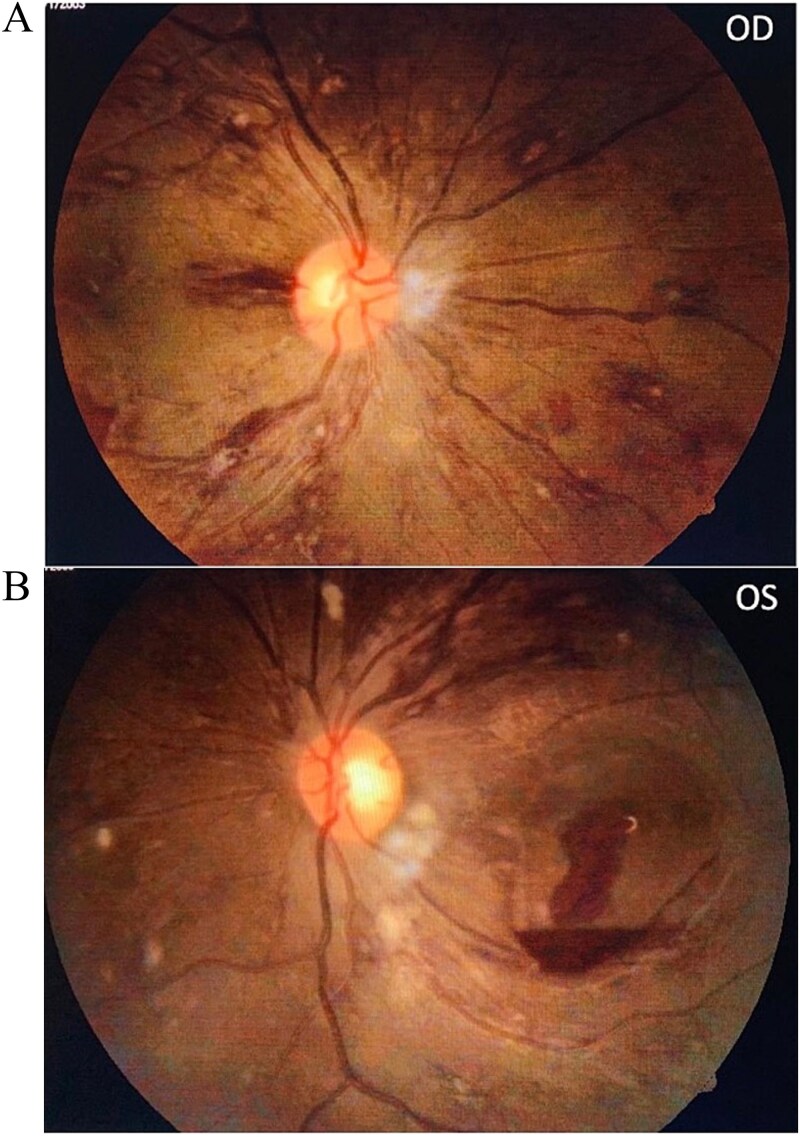
**(A)**: Fundus photo of Right eye showing multiple Roth spots and superficial hemorrhages with venous tortuosity. (B): Fundus photo of left eye showing multiple Roth spots and superficial hemorrhages and sub hyaloid hemorrhages at macula.

On general physical examination patient appeared pale and lethargic. Severe pallor along with knuckle pigmentation was present. Vital signs were stable and no hepatomegaly or splenomegaly were noted. On ocular examination, best corrected visual acuity (BCVA) was 3/60 in both eyes (approximately 20/400 Snellen equivalent), with accurate projection of light in all quadrants. Near vision was reduced to <N36 (unable to read standard near print) with normal color perception. Pupils were equal and reactive to light. Fundus examination revealed multiple Roth spots scattered in the fundus of each eye. Numerous flame-shaped and blot retinal hemorrhages and cotton wool spots were present in the posterior pole along with splinter hemorrhages around the optic disc. Dome-shaped pre-retinal hemorrhage was observed in the macula of each eye, with surrounding retinal edema. The retinal veins were mildly dilated and tortuous. (Fig. 1A and B).

**Figure 2:**
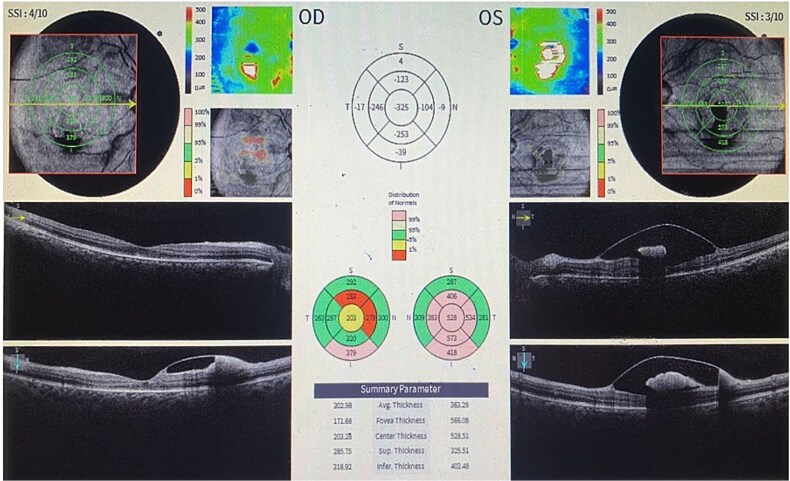
Spectral-domain optical coherence tomography (OCT) of the macula in both eyes confirmed a hyper-reflective collection under the internal limiting membrane (ILM), consistent with a sub-ILM haemorrhage.

Spectral-domain optical coherence tomography (OCT) of the macula in both eyes confirmed a hyper-reflective collection under the internal limiting membrane (ILM), consistent with a sub-ILM hemorrhage; a characteristic ‘double ring’ sign was noted on cross-sectional OCT images. (Fig. 2).

Given the fundoscopic evidence of hemorrhagic retinopathy and general examination, a systemic workup was urgently undertaken. Laboratory investigations revealed severe pancytopenia (Hb 2.3 g/dl, leukocytes 3.0 × 10^9/l, platelets 92 × 10^9/l), macrocytosis (MCV 107 fl), low reticulocyte count, macro-ovalocytes, and hypersegmented neutrophils on peripheral smear. Serum vitamin B12 was 57 pg/ml, folate 3.5 ng/ml, and LDH was elevated. A diagnosis of severe megaloblastic anemia due to vitamin B12 deficiency, compounded by folate deficiency and chronic blood loss, was made.

**Figure 3:**
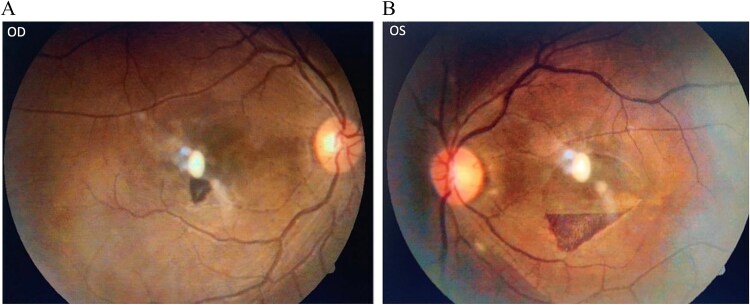
**(A&B)**: Fundus photograph of right and left eye showing resolution of hemorrhages.

**Figure 4:**
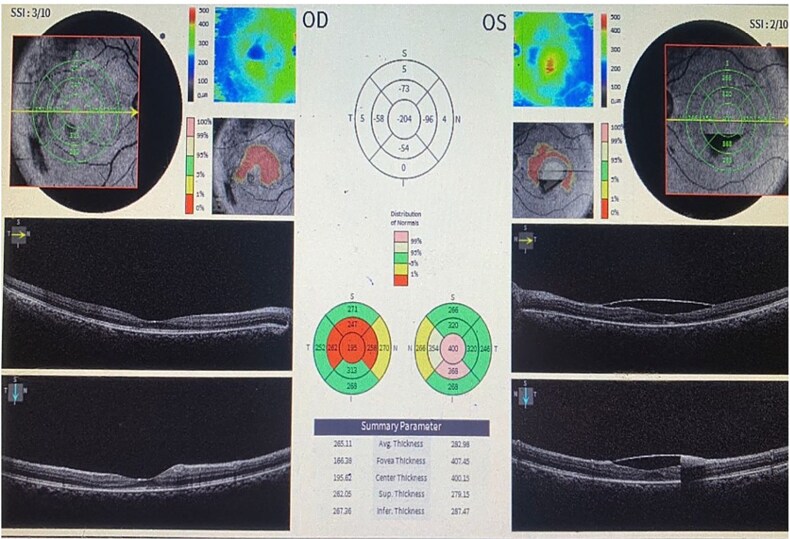
Spectral-domain optical coherence tomography (OCT) of the macula in both eyes showing resolution.

The patient was transfused with four units of packed red blood cells, initiated on intramuscular hydroxocobalamin (1000 μg), oral folic acid, and supportive care. At two weeks, hemoglobin improved to 10.1 g/dl, with normalization of leukocyte and platelet counts. BCVA improved to 6/36 OD and 6/60 OS, with partial clearance of retinal hemorrhages and resolution of cotton wool spots (Fig. 3). OCT confirmed reduction in sub-ILM hemorrhages (Fig. 4) At six weeks, further visual and anatomical recovery was noted.

## Discussion

This case demonstrates an unusual presentation of megaloblastic anemia as an acute bilateral visual loss due to anemic retinopathy. Ophthalmic manifestations of anemia are generally correlated with the severity of the hematological deficit. When hemoglobin falls to critically low levels (approximately less than half of normal), the retinal circulation suffers from hypoxic injury and structural changes. Retinal hemorrhages, soft exudates, venous dilatation, and even optic disc edema (in extreme cases) may occur in various types of anemia [1]. In one study, up to 28% of patients with anemia (especially those with hemoglobin < 8 g/dl) had retinal abnormalities, and the presence of thrombocytopenia significantly raised this risk [1, 4]. Our patient’s hemoglobin of 2.3 g/dl is one of the lowest reported in the context of anemia-related retinal hemorrhages, and she also had moderate thrombocytopenia, together explaining the fundus findings.

The pathophysiology of anemia related retinal changes involves tissue hypoxia and vascular fragility leading to retinal ischemia, which in turn causes cotton wool spots (localized infarcts of the nerve fiber layer) and increased vascular permeability. Hypoxia-induced retinal vascular dilatation and venous stasis, combined with lack of platelet plug production in thrombocytopenia, cause retinal hemorrhages [1].

Bilateral macular hemorrhage and Roth spots as a presentation of megaloblastic anemia have been described only in a handful of case reports [3, 5]. Our case adds to this literature, underlining that megaloblastic anemia should be included in the differential diagnosis when fundus exam reveals unexplained retinal hemorrhages.

In practice, any patient with unexplained retinal hemorrhages and systemic symptoms should prompt a complete blood count. In this case, the ophthalmic examination was pivotal, it prompted immediate laboratory investigations that uncovered life-threatening anemia. This exemplifies the role of ophthalmologists in detecting systemic diseases, a concept highlighted in prior reports where eye findings ‘saved’ patients by leading to timely diagnosis of nutritional anemia [3, 4]. Indeed, the eye can serve as a window to systemic health, and recognition of anemic retinopathy can be vision-saving *and* life-saving.
